# Genomic Characterization of *Enterococcus hirae* From Beef Cattle Feedlots and Associated Environmental Continuum

**DOI:** 10.3389/fmicb.2022.859990

**Published:** 2022-06-27

**Authors:** Sani-e-Zehra Zaidi, Rahat Zaheer, Ruth Barbieri, Shaun R. Cook, Sherry J. Hannon, Calvin W. Booker, Deirdre Church, Gary Van Domselaar, Athanasios Zovoilis, Tim A. McAllister

**Affiliations:** ^1^Lethbridge Research and Development Centre, Agriculture and Agri-Food Canada, Lethbridge, AB, Canada; ^2^University of Lethbridge, Lethbridge, AB, Canada; ^3^Feedlot Health Management Services, Okotoks, AB, Canada; ^4^Cumming School of Medicine, University of Calgary, Calgary, AB, Canada; ^5^Calgary Laboratory Services, Calgary, AB, Canada; ^6^National Microbiology Laboratory, Public Health Agency of Canada, Winnipeg, MB, Canada

**Keywords:** cattle production, antimicrobial resistance, enterococci, genomic signatures, pan-genome

## Abstract

Enterococci are commensal bacteria of the gastrointestinal tract of humans, animals, and insects. They are also found in soil, water, and plant ecosystems. The presence of enterococci in human, animal, and environmental settings makes these bacteria ideal candidates to study antimicrobial resistance in the One-Health continuum. This study focused on *Enterococcus hirae* isolates (*n* = 4,601) predominantly isolated from beef production systems including bovine feces (*n* = 4,117, 89.5%), catch-basin water (*n* = 306, 66.5%), stockpiled bovine manure (*n* = 24, 0.5%), and natural water sources near feedlots (*n* = 145, 32%), and a few isolates from urban wastewater (*n* = 9, 0.2%) denoted as human-associated environmental samples. Antimicrobial susceptibility profiling of a subset (*n* = 1,319) of *E. hirae* isolates originating from beef production systems (*n* = 1,308) showed high resistance to tetracycline (65%) and erythromycin (57%) with 50.4% isolates harboring multi-drug resistance, whereas urban wastewater isolates (*n* = 9) were resistant to nitrofurantoin (44.5%) and tigecycline (44.5%) followed by linezolid (33.3%). Genes for tetracycline (*tetL, M, S/M,* and *O/32/O*) and macrolide resistance *erm(B)* were frequently found in beef production isolates. Antimicrobial resistance profiles of *E. hirae* isolates recovered from different environmental settings appeared to reflect the kind of antimicrobial usage in beef and human sectors. Comparative genomic analysis of *E. hirae* isolates showed an open pan-genome that consisted of 1,427 core genes, 358 soft core genes, 1701 shell genes, and 7,969 cloud genes. Across species comparative genomic analysis conducted on *E. hirae*, *Enterococcus faecalis* and *Enterococcus faecium* genomes revealed that *E. hirae* had unique genes associated with vitamin production, cellulose, and pectin degradation, traits which may support its adaptation to the bovine digestive tract. *E. faecium* and *E. faecalis* more frequently harbored virulence genes associated with biofilm formation, iron transport, and cell adhesion, suggesting niche specificity within these species.

## Introduction

Antimicrobial resistance (AMR) is recognized as one of the major global health challenges of the 21st century. The interconnected microbiomes between humans, animals, and the environment contribute to the emergence, acquisition, and spread of AMR (Hiltunen et al., 2017). A One-health approach provides an in-depth knowledge of the evolution of AMR by focusing on those biological elements that influence the emergence of antimicrobial resistance genes (ARGs) within the microorganism and their dissemination among hosts (human and animals) and the environment (Hernando-Amado et al., 2019). Gram-positive enterococci are core members of the gastrointestinal microbiota of humans and animals and are frequently isolated from soil and water (Byappanahalli et al., 2012; Gilmore et al., 2014). Enterococci often carry ARGs as they compete within complex microbial communities and are exposed to antimicrobials in clinical settings and during livestock production (Murray, 1990; Moreno et al., 2006). Furthermore, depending on the species, enterococci exhibit intrinsic resistance to several antibiotics including cephalosporins, anti-staphylococcal penicillins, aztreonam, aminoglycosides, lincosamides, and streptogramins (Miller et al., 2014). Enterococci are typically commensals, but they can cause nosocomial infections in humans including septicemia, endocarditis, and urinary tract infections (Barnes et al., 2021). There are over 50 species of enterococci with *E. faecalis* and *E. faecium* most frequently linked to human infections. Occasionally, other species including *E. hirae, E. avium, E. durans, E. gallinarum, E. casseliflavus, and E. raffinosus* may also be associated with infections in people (Brayer et al., 2019; Pinkes et al., 2019; Winther et al., 2020). Due to their widespread occurrence and persistence in the environment, enterococci are considered indicators of fecal contamination (Byappanahalli and Fujioka, 2004; Yan et al., 2011) and also serve as key indicator bacteria for AMR surveillance systems in humans and animals (Harwood et al., 2000; Layton et al., 2010).

Studies have indicated that *E. faecium* and *E. faecalis* are more prevalent in human-associated environments, whereas *E. hirae* are prevalent in beef cattle production systems (Zaheer et al., 2020). *E. hirae* only accounts for 1% of enterococcal infections in humans (Heval Can et al., 2020) and is mainly linked to pyelonephritis (Chan et al., 2012; Pãosinho et al., 2016; Nakamura et al., 2021), endocarditis (Talarmin et al., 2011; Pinkes et al., 2019), and biliary tract infections (Tan et al., 2010; Bourafa et al., 2015). As with *E. faecalis* and *E. faecium, E. hirae* infections are typically treated with ampicillin, gentamicin, or vancomycin (Nakamura et al., 2021).

The focus of this study was to investigate the genomic relatedness of *E. hirae* across the environmental continuum and to identify the genetic nature of AMR in *E. hirae*. Furthermore, we applied a pan-genome analysis to identify genes that may account for the predominance of *E. hirae* within beef cattle production systems.

## Methodology

### Bacterial Isolates

**Figure 1:**
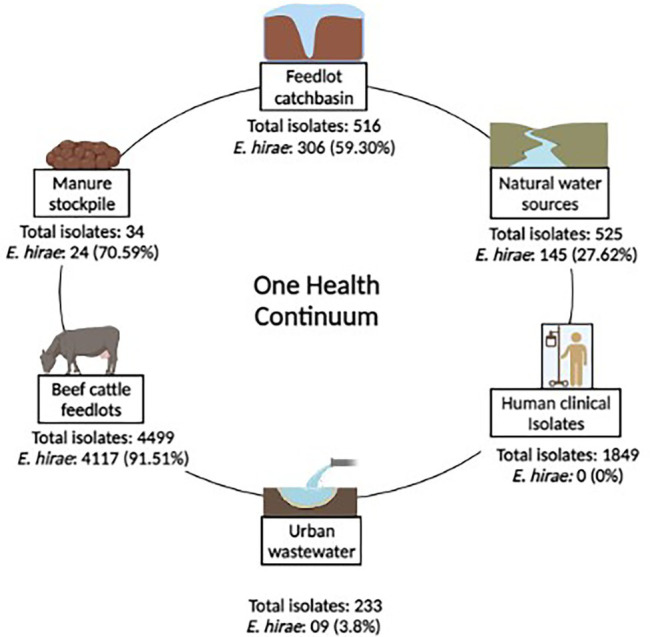
Prevalence of *Enterococcus hirae* isolates (*n* = 4,601) identified across a One-health continuum.

A total of 8,430 *Enterococcus* strains were isolated in a One-Health surveillance study from different segments of the environmental continuum using samples collected from beef production systems (i.e., feedlot cattle feces, catch-basin water, and manure), natural water sources, urban wastewater, and human clinical samples (Zaheer et al., 2020). Bovine fecal samples came from four feedlots in southern Alberta over two years (March 2014–April 2016). Wastewater samples were collected from catch basins that accumulate runoff from the feedlots. Natural surface water samples came from up-stream and down-stream of the feedlots. Urban wastewater samples came from two wastewater plants located in southern Alberta. *Enterococcus* spp. recovered from patients with clinical infections were obtained through the Division of Medical Microbiology, Calgary Laboratory Services (now Alberta Precision Laboratories, Alberta Health Services) (Zaheer et al., 2020). This study focuses on *Enterococcus hirae*, collected as the most prevalent species from beef production system (*n* = 4,601 isolate) (Zaheer et al., 2020). Figure 1 represents the prevalence of *E. hirae* isolates in the sampled sources.

Enterococci were recovered in parallel from two different media types including Bile Esculin Azide (BEA) agar without antibiotic and BEA supplemented with 8 μg/ml erythromycin, followed by species identification. *E. hirae* were identified *via* multiplex PCR targeting groES-EL and muramidase genes (Zaheer et al., 2020). As *E. hirae* was absent among clinical *Enterococcus* isolates (*n* = 1892; Figure 1), complete genomes (*n* = 3) of clinical *E. hirae* were retrieved from NCBI database for comparative genomic analysis (Supplementary Table S1).

### Antimicrobial Susceptibility Testing

Antimicrobial susceptibility testing was performed on a randomly selected subset (*n* = 1319, 29%) of *E. hirae* isolates using the disk diffusion method, as per the Clinical and Laboratory Standards Institute (CLSI) documents M02-A12 and M100-S24. A panel of twelve antibiotics was used for testing based on their common usage for treatment of human enterococcal infections that included those drugs of critical importance (levofloxacin, linezolid, quinupristin/dalfopristin, teicoplanin, vancomycin, and tigecycline), high importance (erythromycin, ampicillin, gentamicin, and streptomycin), and medium importance (nitrofurantoin and tetracycline). *Staphylococcus aureus* ATCC 25923 and *E. faecalis* ATCC 29212 were used as reference quality controls (Zaheer et al., 2020). The BioMic V3 imaging system (Giles Scientific, Inc., Santa Barbara, CA, USA) was used to read zones of inhibition. Isolates were categorized based on CLSI interpretive criteria, except for tigecycline for which EUCAST interpretive criteria (The European Committee on Antimicrobial Susceptibility Testing, 2014) were used.

### Whole-Genome Sequencing

Whole-genomic sequencing of a subset of *E. hirae* isolates (*n* = 286), including isolates originating from bovine feces (*n* = 168), feedlot catch basin (*n* = 62), bovine manure stockpiles (*n* = 8), natural water sources (*n* = 42), and urban wastewater (*n* = 7), was performed using next-generation sequencing technology. Briefly, genomic DNA was extracted using the DNeasy Blood and Tissue Kit (Qiagen, Montreal, QC, Canada) with modifications (Zaheer et al., 2020), followed by DNA quality assessment and quantification using a Nanodrop 2000 spectrophotometer and a Qubit Fluorometer with PicoGreen (Thermo Fisher Scientific, Mississauga, ON, Canada). Isolates were sequenced on an Illumina MiSeq platform using the MiSeq Reagent Kit V3 to generate 2 × 300 bp paired-end reads. Raw read FASTQ files were assessed for the quality of sequence data using FastQC (Galaxy Version 0.72 + galaxy1) (Wingett and Andrews, 2018) and *de novo* assemblies were performed using Shovill (Bankevich et al., 2012). Assembled contigs were then annotated by Prokka to identify all gene-coding sequences (Seemann, 2014).

### AMR Determinants, Virulence, and Plasmid Detection

Assembled genomes were screened for the presence of AMR determinants, virulence genes, and plasmids using ABRicate (https://github.com/tseemann/abricate/) against the NCBI Bacterial Antimicrobial Resistance Reference Gene database (NCBI BioProject ID: PRJNA313047), VirulenceFinder [pmid 15,608,208], and PlasmidFinder databases (Zankari et al., 2012), respectively. Intact prophage were identified using PHASTER tool (Arndt et al., 2016).

### Comparative Genomic Analysis

A total of 289 genomes including 286 assembled genomes from this study and 3 complete *E. hirae* genomes of clinical isolates retrieved from NCBI database (strain: 708, accession: NZ_CP055232.1; strain: FDAARGOS_234, accession: NZ_CP023011.2; and strain: 13344, accession: NZ_CP055229.1) were subjected to phylogenomic analysis. A core-genome phylogenomic tree was constructed using the (SNVPhyl v 1.0) pipeline (Petkau et al., 2017). Briefly, all paired-end reads were mapped against the *E. hirae* reference genome (strain R17; GenBank accession: CP015516.1) to produce read pileups (SMALT v.0.7.5; https://www.sanger.ac.uk/tool/smalt-0/). The read pileups were evaluated for mapping quality (minimum mean mapping quality score of 30), coverage cut offs (15X minimum depth of coverage), and a single-nucleotide variant (SNV) abundance ratio of 0.75 to generate a multiple sequence alignment of SNV containing sites. The final maximum likelihood-based phylogeny was generated by PhyML using unfiltered SNV alignment. Phylogenomic trees and associated metadata were visualized using Interactive Tree Of Life (iTOL) v5 tool (Letunic and Bork, 2021).

Comparative genome analysis was done using the Roary v3.12.0 pipeline with default parameters (Page et al., 2015). Genes identified by Prokka were used to construct pan-genomes. A pan-genome of 289 *E. hirae* isolates was reconstructed to identify core and accessory genes present in *E. hirae*. Furthermore, comparative analysis was performed between *E. faecium* and *E. faecalis,* which are predominantly associated with humans infections, and *E. hirae.* For this purpose, a small subset of *E. hirae* isolates (*n* = 16) representative of the various sources and phylogenetic clades were randomly selected. Similarly, a subset of *E. faecium* (*n* = 26) and *E. faecalis* (*n* = 24) isolates were randomly selected on the same bases from our previous study (Zaheer et al., 2020; BioProject PRJNA604849). The phandango interactive viewer tool (Hadfield et al., 2017) was used to interpret pan-genome data obtained from Roary analysis. This tool utilizes two of the Roary output files: one is a gene absence and presence matrix file that creates a heat map based on the number of genes present or absent in each isolate and a Newick-formatted tree file of accessory genomes used to plot a relatedness dendrogram of the accessory genes present in all isolates.

A pan-genome plot was generated using ggplot2 package of R Studio Version 1.4.1103 (R Studio Inc., Boston, MA, USA) based on two Roary output files (the number of conserved genes and the number of total genes). The number of conserved genes represented the size of the core genome. The number of total genes represented both the core and accessory genomes, creating a curve based on the pan-genome completeness. The pan-genome of an organism is considered “closed” if the curve is predicted to plateau or “open” if the curve is predicted to continue to rise. In contrast to a closed genome, the number of new gene families in an open genome increases with the inclusion of new genomes in the analysis.

Discriminatory genomic signatures between *E. hirae*, *E. faecium,* and *E. faecalis* were identified using Neptune v1.2.5 with default parameter (Marinier et al., 2017). The signature discovery process using Neptune identifies sequences that are sufficiently common to a group of target sequences (inclusion group) and sufficiently absent from non-targets (exclusion group) using probabilistic models. Analyses was done using *E. hirae* genomes as the inclusion group and *E. faecium* and *E. faecalis* as independent exclusion groups, respectively. The genomic signature found in ≥90% of isolates in the inclusion group were selected and annotated using Prokka (Seemann, 2014).

## Results

### Antimicrobial Susceptibility Testing

**Figure 2:**
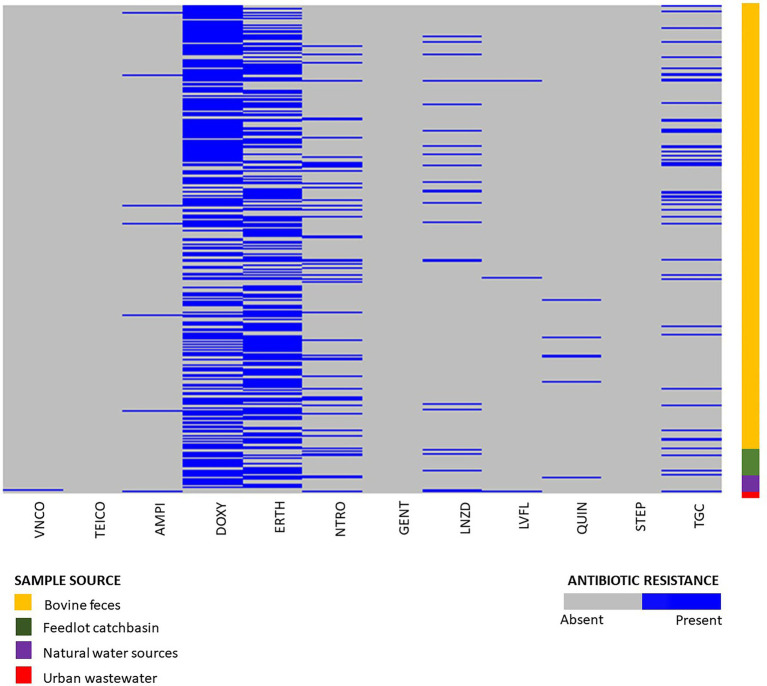
Phenotypic resistance profiles of *Enterococcus hirae* isolated from beef production system (*n* = 1,264) including bovine feces and feedlot catch basin, natural water source (*n* = 45), and urban wastewater sample (*n* = 9).

Phenotypic susceptibility testing was conducted on 1,319 *E. hirae* isolates originating from bovine feces, feedlot catch-basin water, stockpiled bovine manure, and natural and urban wastewater sources. (Figure 2). Fifty-one different resistance profiles were identified with the most frequent being resistant to doxycycline and erythromycin (364/1319, 27.6%) followed by resistance to doxycycline alone (242/1319, 18.3%) (Supplementary Table S2). Across all tested isolates, 14.1% (186/1319) were multi-drug resistant (resistant to ≥3 tested antimicrobials). Antimicrobial susceptibility profiles of all tested isolates are presented in Supplementary Figure S2.

#### Isolates Recovered From BEA Plates Without Erythromycin

Out of 1,319 total isolates tested for antimicrobial susceptibility, 666 isolates were recovered from BEA plates without erythromycin. From these, isolates originating from beef production systems (i.e., bovine feces, catch basin, and stockpiled bovine manure; *n* = 632) exhibited a high prevalence of resistance to tetracycline (376/632, 59.4%), followed by macrolides (200/632, 31.6%), nitrofurantoin (102/632, 16.1%), tigecycline (76/632, 12.0%), linezolid (40/632, 6.32%), ampicillin (9/632, 1.42%), quinupristin/dalfopristin (8/632, 1.26%), vancomycin (1/632, 0.15%), and teicoplanin (1/632, 0.15%) (Supplementary Figure S1).

The natural water source isolates recovered from BEA plates without antibiotics (*n* = 28) also showed a high prevalence of tetracycline resistance (22/28, 78.5%), followed by macrolides (8/28, 28.5%), nitrofurantoin (5/28, 17.8%), and tigecycline (1/28, 3.57%). Resistance against quinupristin/dalfopristin, linezolid, ampicillin, and fluoroquinolones was not detected (Supplementary Figure S1).

Of the 233 *Enterococcus* spp. isolates recovered from urban wastewater (Zaheer et al., 2020), only nine were identified as *E. hirae*. Six of those were recovered from media without erythromycin. Four of the six isolates exhibited resistance to tigecycline (4/6, 66.6%) followed by nitrofurantoin (3/6, 50%), linezolid (3/7, 48.85%), fluoroquinolones (2/6, 33.3%), vancomycin (1/6, 16.6%), and ampicillin (1/7, 14.2%) (Supplementary Figure S1).

#### Isolates Recovered From BEA Plates With Erythromycin

A total of 652 isolates from erythromycin plates were selected for phenotypic antimicrobial testing. Of these isolates, 632 originated from beef production (i.e., bovine feces, catch basin, and stockpiled bovine manure). Tetracycline resistance (437/632, 69%) was the most prevalent resistance in the beef isolates from production systems, followed by macrolides (525/632, 83%), tigecycline (67/632, 10.6%), nitrofurantoin (60/632, 9.5%), linezolid (33/632, 5.2%), quinupristin/dalfoprstin (13/632, 2.0%), ampicillin (9/632, 1.42%), fluoroquinolones (5/632, 0.79%), and gentamicin (1/632, 0.15%) (Supplementary Figure S1).

The isolates recovered from natural water sources (*n* = 17) showed a high prevalence of resistance to macrolides (15/17, 88.2%), followed by tetracycline (13/17, 76.4%), tigecycline (2/17, 11.76%), quinupristin/dalfoprstin (1/17, 5.88%), and nitrofurantoin (1/17, 5.88%). None of the isolates were resistant to linezolid, ampicillin, or fluoroquinolones (Supplementary Figure S1).

A total of three *E. hirae* isolates were recovered from urban waste sources on erythromycin plates. Two of those isolates showed macrolide resistance (2/3, 66.6%), followed by tetracycline (1/3, 33.3%), nitrofurantoin (1/3, 33.3%), quinupristin/dalfoprstin (1/3, 33.3%), and streptomycin (1/3, 33.3%). These isolates were sensitive to teicoplanin, ampicillin, vancomycin, gentamicin, tigecycline, fluoroquinolones, and linezolid (Supplementary Figure S2). Overall, 16.8% of isolates (110/652) recovered from erythromycin plates showed intermediate resistance to erythromycin.

### Whole-Genome Sequencing

Of the *E. hirae* isolates tested for antimicrobial susceptibility, 286 randomly selected isolates were used for whole-genome sequencing. The size of *E. hirae* genomes as interpreted from the assembled sequence read data ranged from 2,307,753 bp to 3,200,875 bp, with a GC content of 36.7%. Detailed assembly statistics are provided in Supplementary Table S3.

### AMR Determinants

**Figure 3:**
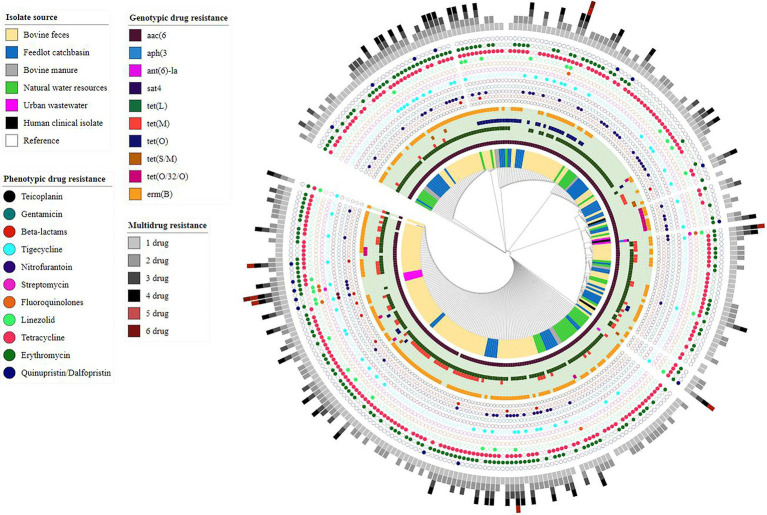
Core-genome phylogenetic tree based on analysis of single-nucleotide polymorphisms (SNPs) of *Enterococcus hirae* genomes (*n* = 291) isolated from different environmental settings including beef production systems and human-related isolates. The genomes were compared using *E. hirae* OG1RF genome (GenBank accession # NZ_CP015516.1/CP015516.1) as a reference.

Assembled genomes (*n* = 286) were screened for the presence of AMR determinants using the Abricate tool along with the NCBI AMR gene database. Ten different ARGs, including aminoglycosides ARGs *aac(6′)-Iid, ant(6)-Ia* and *aph(3)-III*, streptothricin *sat4*, tetracycline ARGs *tet (L, M, O, S/M, (O/32/O))*, and macrolide ARG *erm(B)* were identified across the examined genomes (Figure 3). Overall, nineteen different resistance genotypes were identified with the most frequent being *aac(6′)-lid-tet(L)-erm(B)* (87/286, 30.41%) followed by *aac(6′)-lid-tet(L)-tet(M)-erm(B)* (64/286, 22.37%) (Supplementary Table S4). The aminoglycoside resistance gene *aac(6′)-lid* was identified in all but two of the *E. hirae* genomes (284/286, 99.30%).

Of the 286 sequenced isolates, 238 were recovered from beef production systems (i.e., bovine feces, feedlot catch basin, and stockpiled bovine manure). *tet(L)* (199/238, 83.61%) was the most prevalent ARG identified in these isolates, followed by *erm(B)* (179/238, 75.21%) and the tetracycline resistance genes, *tet (M)* (73/238, 30.67%), *tetO* (36/238, 15.12%), *tet(O/32/O)* (13/238, 5.46%), and *tet(S/M)* (03/238, 1.26%).

Similar to beef production system isolates, *E. hirae* isolates recovered from natural water sources located near feedlots showed a high prevalence of *tet(L)* (38/41, 92.68%) followed by *erm(B)* (27/41, 65.85%). Occasionally, *tet(M)* (4/41, 9.75%), *tet(O)* (4/41, 9.75%), and tet(O/32/O) (2/41, 4.87%) were also present in these isolates.

Among seven *E. hirae* isolates recovered from urban wastewater, the streptomycin resistance gene *ant(6)-la* was present in two isolates (2/7, 28.57%). The kanamycin/neomycin *aph(3′)-III* and streptothricin *sat4* resistance genes were both found in a single urban wastewater isolate (1/7, 14.28%). Tetracycline resistance gene(s) were not found in any of these isolates, whereas *erm(B)* was only detected in one isolate (1/7, 14.28%).

*tet(L)* and *erm(B)* were found together in 63.63% of total isolates (182/286) indicating a strong correlation. Similarly, *tet (L)* and *tet (M)* coexisted in 24.12% of isolates (69/286) and in most cases were found on the same contig (60/69, 87%) in assembled genomes.

*E. hirae* genotypes generally associated with quinolone resistance (i.e., presence of quinolones resistant gene (qnr) or DNA gyrase and DNA topoisomerase IV genes mutations) and linezolid resistance (i.e., mutations in the 23S ribosomal RNA gene or presence of resistance genes including *cfr*, *cfrB*, *optrA,* and *poxtA*) were not identified.

### Virulence Factors

Within the 286 *E. hirae* isolates, we identified nine different virulence genes associated with biofilm formation *(bopD)*, capsular polysaccharides biosynthesis *(cpsA, cpsB, and cap8E)*, hyaluronic acid production (*hasC*), proteolytic activity/chaperones *(clpP),* fibrinogen adhesions protein *(fss3)*, bile salt hydrolase *(bsh)*, and listeria adhesion protein *(lap).* All isolates carried *cap8E*, *clpP*, *cpsA*, *cpsB*, *bopD*, and *lap* genes, while *hasC*, *bsh,* and *fss3* were found in 98.95% (283/286), 89.86% (257/286), and 2.44% (7/286) of total isolates, respectively. These genes were also identified in publicly available clinical *E. hirae* genomes from humans. Pili protein-encoding gene *ebpC* was only detected in one of the human clinical isolates retrieved from NCBI. Detailed information is provided in Supplementary Table S5.

### Plasmid Identification

Among all *E. hirae* isolates (*n* = 286), 16% carried plasmids. Seven different plasmids (rep1, rep2, rep11, rep17, rep18, repUS7, and repUS12) were identified. Among these, rep2 and rep17 were recovered from all sample types except urban wastewater. In contrast, rep1, rep18, and repUS7 were recovered from urban wastewater samples. Two out of fifteen rep17 plasmids carried *erm(B)*, whereas one out of twenty rep2 plasmids carried *tet(L).* Of six repUS12 plasmids, five carried *tet (L)* and were recovered from bovine feces.

### Prophage Identification

A total of 30 genomes were randomly selected from all sample sources to identify bacteriophage using PHASTER. All isolates contain at least one prophage ranging from 7 to 48 kb in size. Twenty-four intact prophage sequences were identified, with 95.5% identified as members of the family Siphoviridae. None of the identified prophages harbored ARGs.

### Comparative Genomic Analysis

Core-genome phylogenomic analysis was conducted on the 286 *E. hirae* isolated in this study and the three *E. hirae* genomes retrieved from NCBI. The *E. hirae* isolates clustered into six different clades, with no obvious segregation by source (Figure 3).

**Figure 4:**
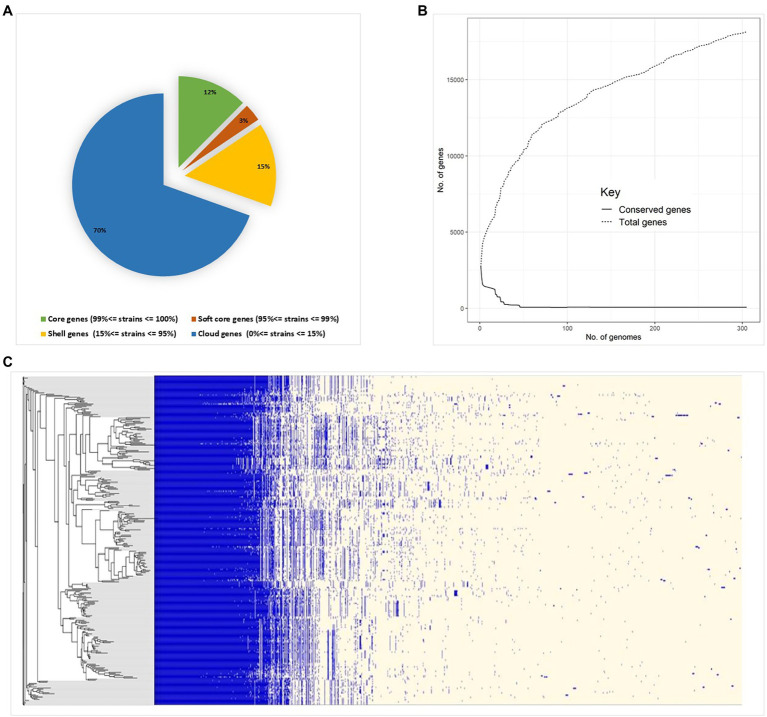
Pan-genome estimation of *Enterococcus hirae* **(A)** genomic statistics and pan-genome estimation of 291 isolates. **(B)** Development of pan- and core genomes, illustrating the open nature of the pan-genome. **(C)** Heat map representing absence or presence of genes in isolates and the phylogenetic genetic tree generated from accessory genes.

**Figure 5:**
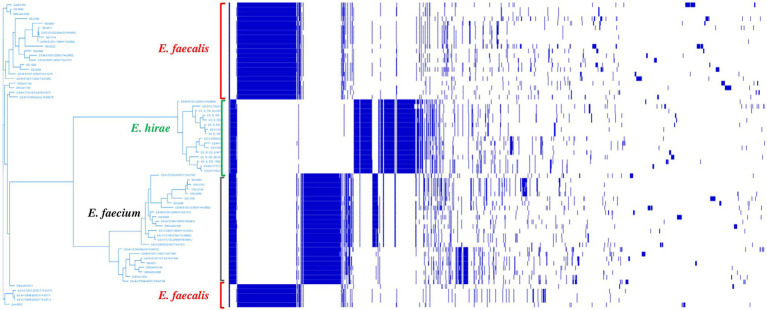
Heat map representing absence or presence of genes and phylogenetic genetic tree generated from accessory genes of *Enterococcus hirae* (*n* = 16), *Enterococcus faecium* (*n* = 26), and *Enterococcus faecalis* (*n* = 24).

Pan-genome analysis of *E. hirae* isolates identified 1,427 core genes (99 to 100% of strains), 358 soft core genes (95 to 99% of strains), 1701 shell genes (15 to 95% of strains), and 7,969 cloud genes (0 to 15% of strains) (Figure 4A). The pan-genome of *E. hirae* is open as the number of accessory genes progressively increased with increasing genomes (Figure 4B). In addition, the gene presence and absence heat map showed that the accessory genome constituted a large part of the pan-genome, indicative of a high level of genomic diversity within this species (Figure 4C). Cross-species comparative analysis of *E. hirae*, *E. faecium,* and *E. faecalis* genomes, highlighted the genomic diversity within *Enterococcus* spp. as the total core genome shared between three species was small (Figure 5). Furthermore, it also demonstrated the distinct genomic traits of each species as illustrated by the gene absence and presence heat map (Figure 5).

A total of 1,069 discriminatory genomic signatures were recognized in *E. hirae* compared to *E. faecalis* (*n* = 808) and *E. faecium* (*n* = 261). Most of these genomic signatures encoded for unknown hypothetical proteins (454/1069, 42.46%).

Neptune analysis is capable of identifying inter-species genomic variation, as discriminatory loci were identified in all three species. These loci coded for genes required for the synthesis of aromatic amino-acids including chorismate synthase (*aroC*), cyclohexadienyl dehydrogenase (*tyrC*), genes for sugar transport including permease (*yteP*), transcriptional regulator (*mtlR)*, lichenan permease IIC component (*licC*), and lipoprotein (*lipO*).

Signatures found exclusively in *E. hirae* genomes included genes associated with the phosphotransferase system for galactitol (*gatA, B,* and *C*) and fructose (*fruA, frwA,* and *frwD*), peptidoglycan synthesis (*rodA ftsW, mur E, F, J,* and *Y*), teichoic acid synthesis (*tag H, G,* and *U*), coenzyme A biosynthesis (*coaD*), vitamin B12 synthesis (*nrdZ*), chitin degradation (*chiA*), capsule synthesis (*epsE, pglF, ywqD,* and *ywqC*), riboflavin synthesis (*ribBA*, *D, E,* and *H*), vitamin B6 synthesis (*yvgN*), vitamin uptake transporter (*queT*), gamma-aminobutyric acid (GABA) production (*glsA2, gadC,* and *amt*), cardiolipin biosynthesis (*clsA*), bacitracin export (*bceA* and *B*), xenobiotic degradation (*nylA*), and iron transport (*yqgN, feuC, feuB, fepC,* and *yfiY*).

Furthermore, genes that may be associated with antimicrobial resistance were also identified in *E. hirae* genomes such as those encoding for multi-drug transporters (*marA, mepE msrR,* and *yxlF*), doxorubicin resistance (*drrA*), sulfonamide resistance (*bcr*), and penicillin-binding protein (*pbp*). Early secreted antigenic target (ESAT) system genes (*eccC, essB*, and *esxA*) were also found in *E. hirae* genomes.

Compared to *E. hirae*, 160 and 944 discriminatory genomic signatures were identified in *E. faecium* and *E. faecalis* genomes, respectively, with most of these genes associated with various aspects of cellular metabolism, such as molybdopterin biosynthesis (*mog*, *modB,* and *modB*); cadmium, zinc, and cobalt transport (*cadA*); copper export (*copY* and *copA*); phosphotransferase system for glucitol/sorbitol (*srlA*, *srlE,* and *srlB*), sorbose (*sorB*, *sorF,* and *sorA*), mannose (*manX* and manZ), cellobiose (*celA*), mannitol (*mtlA*, *mtlF,* and *mtlD*), maltose (*malX*), ascorbate (*ulaC* and *ulaA*), and beta-glucosides (*bglF*). In addition, genes encoding *E. faecalis* and *E. faecium* pathogenesis were also identified, such as biofilm formation genes (*brpA*, *icaA,* and *lytR*); virulence genes, including unsaturated chondroitin disaccharide hydrolase (*ugl*); anthrax toxin regulator positive (*atxA*); hemin transport system (*hmuU* and *hmuT*); sialic acid TRAP transporter small permease (*siaQ*); carnitine transport system (*opuCB* and *opuCA*); arginine/ornithine system (*argR* and *arcD1*); genes encoding the adhesions, including gelatinase (*gelE*) and collagen (*cna*); and genes encoding for antimicrobial resistance, including penicillin-binding protein (PbpE, PbpX, and PbpF), multi-drug resistance protein (YkkC, YkkD, and Stp), tetracycline repressor protein (TetR), fluoroquinolones export protein (Rv2688c), and macrolide export protein (MacB) (Supplementary Table S7).

## Discussion

Enterococci are ubiquitous Gram-positive bacteria. They colonize gastrointestinal tracts of most multicellular eukaryotic organisms including humans, animals, and insects and aide in digestion and gut metabolic pathways (De Graef et al., 2003; Farrow and Collins, 1985; Devriese et al., 1990; Andrew and Mitchell, 1997; de Vaux et al., 1998; Muniesa et al., 1999; Fogarty et al., 2003; Law-Brown and Meyers, 2003; Maria da Gloria et al., 2006; Layton et al., 2010; Giraffa, 2014). In addition, they are also found in food, plant, and water ecosystems (Müller et al., 2001; Svec et al., 2001; Klein, 2003; Švec et al., 2006; Byappanahalli et al., 2012). Enterococci are remarkably resilient to broad pH ranges, temperature variation, and osmotic pressure, traits that contribute to their broad distribution in nature (Heim et al., 2002; Caretti and Lubello, 2003; Anderson et al., 2005). Resistant bacterial populations are selected by the exposure of commensal gut microorganisms such as enterococci to antimicrobials that are used for disease treatment and prevention (Francino, 2016). The ubiquitous nature of enterococci may facilitate the dissemination of antimicrobial resistance genes between different environments. For this reason, it is imperative to identify antimicrobial resistance determinants and their role in the spread of antimicrobial resistance (Cameron and McAllister, 2016). Here, we focused on *E. hirae* isolates recovered from a One-Health surveillance study (Zaheer et al., 2020). The genomic relatedness of *E. hirae* was examined across various sampling matrices of the continuum and AMR determinants that contribute to antimicrobial resistance were identified. Furthermore, we examined the genomic traits of *E. hirae* that may facilitate their growth in the cattle gut as compared with other human-associated *Enterococcus* species.

As described previously, *E. hirae* is highly prevalent in cattle and thus can be readily isolated from bovine feces, bovine manure, and feedlot catch-basin water samples (Jackson et al., 2011; Zaheer et al., 2013, 2020; Beukers et al., 2015). The number of *E. hirae* isolates recovered from urban wastewater was low (3%) and most of the *Enterococcus* spp. from this source were identified as either *E. faecalis* or *E. faecium*. Similarly, only *E. faecalis* or *E. faecium* was identified among human clinical isolates, confirming that *E. hirae* is generally not associated with human infections. However, *E. hirae* have occasionally been isolated from human cases of septicemia (Gilad et al., 1998), endocarditis (Poyart et al., 2002; Talarmin et al., 2011), urinary tract infections (Chan et al., 2012; Bourafa et al., 2015), spondylodiscitis (Canalejo et al., 2008), and acute pancreatitis (Dicpinigaitis et al., 2015). The rarity of this species among clinical enterococci isolates suggests that this species may not be as virulent as *E. faecalis* and *E. faecium*. This finding is also evident from our comparative genomic analysis where virulence genes were frequently identified in *E. faecalis* and *E. faecium,* but not in *E. hirae*.

The phenotypic resistance profiles of 1,319 *E. hirae* isolates showed that antimicrobial use and resistance phenotype were linked within a particular environment. For example, macrolides and tetracyclines are commonly used in beef cattle production systems for disease treatment and prevention including prophylaxis/metaphylaxis (Hurd and Malladi, 2008; Cameron and McAllister, 2016; Vikram et al., 2017). Isolates recovered from bovine feces, feedlot catch basin, stockpiled bovine manure, and natural surface water in the vicinity of the feedlots showed high occurrence of resistance to these antibiotics. Although only nine *E. hirae* isolates were recovered from urban wastewater, their resistance profiles indicated linkage with drugs commonly used to treat human infections including nitrofurantoin, tigecycline, and linezolid.

Genotypic resistance profiles of *E. hirae* corroborated to their phenotypic profiles, where tetracycline and macrolide resistance genes were predominantly present in isolates recovered from the beef cattle production system and natural water sources. This is consistent with previous studies where tetracycline and macrolide resistance genotypes were prevalent in beef production systems (Zaheer et al., 2013, 2019; Rovira et al., 2019). Tetracycline resistance was associated with the presence of *tetL, M,* and *O*. Two mosaic tetracycline genes *tetS/M* and *tetO/32/O* were also identified. *tetL* confers resistance *via* an efflux mechanism, while *tetM*, *tetS/M*, O, and *O/32/O* encode for ribosomal protection proteins (Safferling et al., 2003; Kazimierczak et al., 2008; Barile et al., 2012; Crespo et al., 2012; Dönhöfer et al., 2012). These genes are mostly found on transposable elements that are often linked with chloramphenicol and macrolide resistance determinants (Opal and Pop-Vicas, 2015). Macrolide resistance was associated with the presence of *erm(B),* which confers resistance against macrolide-lincosamide-streptogramin antibiotics (Okitsu et al., 2005). Others have also found *erm(B)* in *E. hirae* (Portillo et al., 2000; Chajęcka-Wierzchowska et al., 2016), as well as in *E. faecalis* and *E. faecium* isolated from chickens (Kim et al., 2019, 2021), turkies (Tremblay et al., 2011; Demirgül and Tuncer, 2017), pigs (Aarestrup, 2000), fermented food (Sanchez Valenzuela., 2013), and clinical settings (Schmitz et al., 2000; Chen et al., 2010; Wang et al., 2021). The macrolide resistance gene and tetracycline resistance genes in *E. hirae* appear to be identical to those in *E. faecalis* and *E. faecium* (Beukers et al., 2017; Zaheer et al., 2020). Considering that all of these species carry similar AMR determinants, the possibility of horizontal gene transfer across species seems probable (Palmer et al., 2010). Studies have identified the presence of pheromone responsive plasmids in *E. faecium* and *E. faecium* that either encode vancomycin resistance or facilitate the transfer of plasmids carrying vancomycin ARGs into recipient cells (Flannagan and Clewell, 2002; Johnson et al., 2021). These plasmids can also transfer between *Enterococcus* species, as the pMG1 plasmid has been shown to transfer between *E. faecium* and *E. faecalis*, and from *E. faecium* to *E. hirae* (Costa et al., 1993).

Aminoglycoside gene *acc(6′)-lid* is known to be intrinsic in *E. hirae* (Costa et al., 1993) and was detected in all but two genomes, likely as a result of gene coverage and assemblage issues. It is not surprising that vancomycin resistance genes were not identified in *E. hirae*, as this drug is not approved for veterinary use in North American cattle. Our result is consistent with a previous study where vancomycin resistance genes were not identified in *Enterococcus* spp. isolated from bovine feces (Beukers et al., 2017). The virulence genes identified in *E. hirae* were mostly associated with biofilm formation and polysaccharide biosynthesis, as described by others (Hashem et al., 2017). The 10 virulence genes that were identified in *E. hirae* were similar to those in *E. faecalis* and *E. faecium*, but many more (i.e., 49) virulence genes were found in *E. faecium* and *E. faecalis* (Zaheer et al., 2020).

Pan-genome analysis is an important comparative analysis tool that allows linkages between genetic changes and specific phenotypes as it describes core- and accessory genomes as well as species-specific genes (Vernikos, 2020). The core genome constituted only 64% of the total genome in *E. hirae*. Both horizontal and vertical transfer of genes, including those that confer antimicrobial resistance, play a significant role in shaping the pan-genome of a bacterial species (Ding et al., 2018). The pan-genome of *E. hirae* was considered “open” as there was no sign of saturation and it would be expected to expand with the addition of new genomes as illustrated by the pan-genome curve (Figure 4B). The high presence of cloud genes reflects the heterogeneity of the pan-genome of *E. hirae.* The existence of *E. hirae* in diverse environments may increase the chance of gene acquisition, in contrast to other *Enterococcus* species that may live in more specific environments that require less genomic variation for survival (Costa et al., 2020).

Gram-positive bacteria have sophisticated cell wall structures that ensure bacterial structural integrity and cellular viability and are also a major component of the host defense system (Koch, 2003; Silhavy et al., 2010). For this reason, numerous studies have been conducted to explore components of cell wall synthesis pathways as potential targets for drug therapy. Genes involved in cell wall synthesis were identified as discriminatory genomic signatures between *E. faecalis* and *E. hirae*. Identification of these different signatures [peptidoglycan synthesis genes (*Mur E, F,* and *Y*), penicillin-binding protein (*pbpE* and *pbpX*), teichoic acid synthesis genes (*tag H, G,* and *U*), and enterococcal polysaccharide antigen (*eps E, D, M,* and *N*)] may identify targets that offer more specific drug development against *E. faecalis* and *E. faecium* (Parisien et al., 2008).

Members of gut microflora compete with each other for nutrient availability. Therefore, the ability of one bacterial species to utilize multiple nutrients for energy generation provides an advantage over other species. We found genes involved in the synthesis of cobalamin (vitamin B12), pyridoxine (vitamin B6), riboflavin (vitamin B2), biotin (vitamin B7), and folic acid exclusive to the *E. hirae* species compared with other enterococci analyzed in this study. With vitamins being undeniably important for both bacteria and the mammalian host, gut bacteria associated with their production directly contribute to the development and welfare of the host and thus may have a specific function within the microbiome of the digestive tract of cattle. In *E. hirae* genomes, multiple phosphotransferase systems (PTS) for fructose, galactitol, mannose, sorbose, glucose, N-acetyl glucosamine, and cellobiose were identified. The presence of these PTS promotes colonization of these bacterial populations (Jeckelmann and Erni, 2020). In addition, compared to *E. faecalis* and *E. faecium*, *E. hirae* harbored genes that were predicted to contribute to the synthesis of bacterial cellulose. These findings indicate that cellular metabolism genes identified in *E. hirae* may contribute to fitness within the cattle gut, accounting for its high prevalence in beef cattle.

Analysis of the annotated genomes indicated that *E. hirae* possessed genes coding for the production of antimicrobial agents like bacilysin, subtilosin, and narbonolide. Bacilysin is a dipeptide antimicrobial with antifungal and antibacterial activity (Özcengiz and Öğülür, 2015). Subtilosin belongs to the lantibiotics class of bacteriocins and has anti-biofilm activity (Babasaki et al., 1985; Algburi et al., 2017). These bacteria are also capable of producing gamma-aminobutyric acid (GABA), an inhibitory neurotransmitter. GABA may increase feed intake in cattle and reduce anxiety and pain (Sarasa et al., 2020; Mamuad and Lee, 2021). These findings suggest that *E. hirae* may have probiotic properties that could benefit the gastrointestinal environment of cattle (Ben Braïek and Smaoui, 2019). Previously, *E. hirae* has been employed as a probiotic bacteria in freshwater fish (Adnan et al., 2017). Recent studies have also demonstrated that *E. hirae* may confer probiotic properties within the intestinal tract of cattle (Arokiyaraj et al., 2014; Daillère et al., 2016).

One of the goals of this study was to identify the niche-specific genes in *E. faecalis* and *E. faecium* that may contribute to virulence and infection. Several virulence factors that contribute to the pathogenesis of *E. faecalis* and *E. faecium* have been reported (Ali et al., 2017; Zhou et al., 2020). Several virulence genes were unique to *E. faecium* and/or *E. faecalis* and were not found in *E. hirae*. Members of the SlyA/ MarA family of proteins are associated with virulence gene regulation, promote biofilm formation, and act as cell adhesions. The presence of genomic signatures corresponding to these genes may in part account for the higher prevalence of *E. faecalis* and *E. faecium* infections than *E. hirae* infections in humans (Mäkinen et al., 1989; Michaux et al., 2011; Yang et al., 2015). Lipoproteins facilitate intake of nutrients and are often associated with ABC transporters that are linked to pathogenesis. This supports our findings as genes encoding lipoproteins mapped with ABC transport systems for manganese, arabinose, and methionine (Zhang et al., 1998; Hutchings et al., 2009). Furthermore, in *E. faecalis* and *E. faecium,* we also identified an arginine-ornithine antiporter which could contribute to cell fitness by facilitating arginine uptake. A study conducted to investigate the role of arginine-ornithine antiporter in *Streptococcus suis* reported that intercellular survival of this pathogen within epithelial cells was compromised in the absence of the antiporter (Fulde et al., 2014).

The potential of *E. hirae* as an opportunistic pathogen cannot be ignored, as it is occasionally recovered from both human and animal clinical samples (Nicklas et al., 2010; Dicpinigaitis et al., 2015; Ebeling and Romito, 2019; Pinkes et al., 2019; Bilek et al., 2020). Despite a higher prevalence of virulence genes in *E. faecium* and *E. faecalis,* some virulence genes were also identified in *E. hirae,* like genes associated with the ESX (or Type VII) secretion system, bicyclomycin resistance, capsule biogenesis, quorum sensing system, and an ABC transporter for iron import (Stauff et al., 2008; Rutherford and Bassler, 2012; Borst et al., 2015; Hatosy and Martiny, 2015; Poweleit et al., 2019). In addition, a lipoprotein gene associated with the iron transport system has been identified and is thought to play a role in *E. hirae* establishing opportunistic infections (Hutchings et al., 2009).

In conclusion, *E. hirae* has a tremendous ability for survival and adaptation. It has acquired resistance to the most common antimicrobials used in beef production systems. In addition, cellular metabolism genes involved in vitamin biosynthesis, multiple ABC and PTS transport systems, chitin degradation, and cellulose synthesis provide selective advantage and facilitate intestinal colonization of the cattle gut. As *E. hirae* appears to be uniquely adapted to cattle hosts, this likely limits the extent to which it transfers genes to bacteria that are important in human health. Regardless, the absence of resistance to critical antimicrobials in *E. hirae* gives credibility to limiting use of these drugs in feedlots and suggests that prudent management of antimicrobials in feedlot settings is an important practice.

## Data Availability Statement

The datasets presented in this study can be found in BioProject PRJNA604849 and in online repositories. The names of the repositories and accession number(s) can be found in the article/supplementary material.

## Ethics Statement

The animal study was reviewed and approved by the Lethbridge Research Centre Animal Care and Use Committee and was conducted according to the Canadian Council of Animal Care Guidelines. Sampling procedures were conducted according to the protocol approved by the Animal Care Committee, University of Calgary (Protocol ID: AC14 -0029). Written informed consent was obtained from the owners for the participation of their animals in this study.

## Author Contributions

RZ and TM designed the study. SH and CB arranged for collection of feedlot samples, metadata, and antimicrobial use data. RB, SC, and RZ isolated and characterized enterococci. RB performed AST. S-e-ZZ, generated figures, analyzed overall data/results, and wrote first draft of the manuscript. GD provided and managed the bioinformatics cluster facility and bioinformatics tools. S-e-ZZ and RZ analyzed sequence data. TM and AZ provided funding and supervision. All authors participated in editing and reviewing the manuscript and approved the final manuscript.

## Funding

Authors are grateful to the Major Innovation Fund of the Government of Alberta in conjunction with the University of Calgary AMR One Health Consortium, the Beef Cattle Research Council (BCRC) Project FOS 10.13, and Genomics Research and Development Initiative of the Government of Canada for financial support.

## Conflict of Interest

CB is part owner and managing partner of Feedlot Health Management Services.

The remaining authors declare that the research was conducted in the absence of any commercial or financial relationships that could be construed as a potential conflict of interest.

## Publisher’s Note

All claims expressed in this article are solely those of the authors and do not necessarily represent those of their affiliated organizations, or those of the publisher, the editors and the reviewers. Any product that may be evaluated in this article, or claim that may be made by its manufacturer, is not guaranteed or endorsed by the publisher.
